# Quantifying and mapping uncertainty in urban sentiment prediction: a combined approach with entropy and SHAP explanations

**DOI:** 10.3389/fpubh.2026.1796565

**Published:** 2026-06-03

**Authors:** Iuria Betco, Ana Isabel Ribeiro, Cláudia M. Viana, Jorge Rocha

**Affiliations:** 1 Centre of Geographical Studies, Institute of Geography and Spatial Planning, University of Lisbon, Lisbon, Portugal; 2 CEGOT—Centre of Studies in Geography and Spatial Planning, Faculty of Arts and Humanities of Porto, University of Porto, Porto, Portugal; 3 EPIUnit ITR, Instituto de Saúde Pública da Universidade do Porto, Universidade do Porto, Porto, Portugal; 4 Associate Laboratory Terra, Lisbon, Portugal

**Keywords:** agnostic models, artificial intelligence, sentiment analysis, spatial uncertainty, urban environment

## Abstract

**Introduction:**

Mental health issues have been increasing globally, possibly linked to urbaniza¬tion and related lifestyles. There’s a growing awareness that different aspects of the urban environment can influence mental health, either enabling or restrict¬ing behaviors that affect well-being. Recent studies have increasingly used social media data and machine learning techniques to assess urban well-being. However, these approaches often lack interpretability and do not explicitly account for prediction uncertainty, limiting their reliability for spatial decision-making.

**Methods:**

This study uses sentiment labels derived from georeferenced posts on X (formerly Twitter) via the NRC Emotion Lexicon. Supervised machine learning models are trained to predict sentiment based on urban environmental variables and generate spatial predictions for Lisbon, with performance compared across K-Nearest Neighbour (KNN), Random Forest (RF), Extreme Gradient Boosting (XGBoost), and Neural Network (NN). To go beyond simple point predictions, uncertainty at each location is measured with Shannon Entropy, based on class probabilities. Post-hoc explanations using SHapley Additive exPlanations (SHAP) identify urban features most impacting sentiment predictions.

**Results and discussion:**

Uncertainty maps combined with SHAP outputs show where interpretations are more reliable (low uncertainty) and where caution is needed (high uncertainty). In Lisbon 2019, SHAP identified proximity to cycling networks, fitness facilities, and NDVI as key factors influencing sentiment. This framework enhances spatial understanding of sentiment-environment relationships and offers a transparent way to identify areas where predictions are more reliable.

## 1 Introduction

The city can be viewed as a dynamic interaction between humans and the environment (1, 2). This relationship influences daily experiences, behaviors, and choices, affecting both physical and mental health. While individual health is mainly influenced by social and economic factors, environmental factors and health behaviors are also significant. Behaviors are personal decisions, but environments can promote or trigger specific actions (3). For instance, access to parks encourages physical activity and is linked to lower stress and better mood, while pollution exposure can cause health issues like asthma and is associated with depression and anxiety (3–7). In this framework, subjective sentiments are key indicators of quality of life and mental health, and improving the built environment may foster better residents’ well-being (8).

Although sentiment analysis is important, capturing subjective sentiments in real time remains difficult. Many studies depend on questionnaires, which have limitations like limited quantitative data, coverage issues, data-collection challenges, and problems reproducing results (8). As an alternative, social media data are increasingly used to evaluate population sentiments, emotions, and signals related to mental health (9). Compared to questionnaires and interviews, social media data provide larger samples for sentiment analysis (8) and can aid decision-making aimed at creating healthier, more innovative, and sustainable cities (10). As a result, sentiment analysis has become a common method to explore how online-shared sentiments relate to urban environments (2, 11). However, despite rapid growth in research and data availability, there is still limited evidence from big data on how sentiments vary across space and time and what factors drive them (2). To address this gap, it is necessary to develop approaches that can link sentiment patterns to urban environmental characteristics in a spatially explicit way, while accounting for the complexity and variability of these relationships.

Empirical and traditional statistical techniques like principal component analysis, clustering, regression, and other linear methods have frequently been utilized (12). While these approaches can offer valuable insights for effective planning, management, and decision-making, they do have certain analytical limitations. Specifically, they often fail to fully capture nonlinear behaviors or account for spatial heterogeneity and autocorrelation effects (13–15). These issues are especially significant when modeling complex phenomena such as sentiment expression across diverse urban settings. Machine Learning (ML), a branch of artificial intelligence (AI), has been increasingly used to tackle these issues and is often reported to match or surpass traditional methods in predictive accuracy (16–18). ML can handle diverse data types, structures, and large volumes (big data) (19), and it tends to be less affected by variable scaling (12), making it easier to combine data from various sources to model complex nonlinear relationships that explain sentiment changes in urban settings. However, many ML algorithms remain treated as “black boxes,” complicating the interpretation of how predictions are generated (19). These benefits are especially important in urban sentiment research, where predictors are often numerous, varied, and spatially arranged.

Machine learning methods have gained popularity because of their ability to identify patterns in complex, high-dimensional data (20). High-dimensional data are characterized by having a number of features per observation that is similar to or greater than the number of observations (21). These methods can model both categorical and continuous response variables. However, unlike traditional parametric methods such as multiple regression, they do not easily provide information about prediction error, like the standard error of prediction for a new data point (22).

Explainable Artificial Intelligence (xAI) has become a crucial research area, providing statistical and visualization tools that improve the interpretability of machine learning models (19, 23, 24). Model-agnostic methods are introduced as interpretation techniques to explain the underlying functions driving the entire behavior of ML models (19, 24, 25). The main benefit of a model-agnostic approach is its versatility, as it can interpret any type of black-box ML model, which is vital when ML outputs influence decision-making processes (25).

The model-agnostic approach offers explanations based on the varied behaviors of complex fitted models, providing insights at both global and local levels (12). Typically, global techniques like Permutation Feature Importance (PFI) and Partial Dependence Plot (PDP) describe the overall behavior of a machine learning model, usually as expected values derived from the data distribution. In contrast, local interpretation methods such as Local Interpretable Model-Agnostic Explanations (LIME) and Shapley Additive ExPlanations (SHAP) focus on explaining individual predictions (19, 26). It is important to remember that local fidelity does not equate to global fidelity; features that are globally significant may not be relevant in specific local contexts, and vice versa (26). Depending on the analysis goal, different methods can be combined to interpret the same model either globally or locally (12).

However, interpretability alone does not resolve a key challenge in spatial prediction: uncertainty. When estimating a variable at a specific location, some degree of uncertainty is inevitable, and “prediction uncertainty” indicates our confidence level in the model’s output (27). While many studies have compared geostatistical and machine learning methods based on point prediction accuracy, fewer have evaluated the trustworthiness of their uncertainty estimates, especially in spatial contexts (22, 28, 29). For example, Coulston et al. introduced a method to estimate prediction uncertainty in Random Forest regression models within a spatial framework (22). Kirkwood et al. analyzed how well Ordinary Kriging and Quantile Regression Forests can produce reliable prediction uncertainties for geochemical mapping in southwest England (28). Similarly, Vaysse and Lagacherie compared these approaches for French digital soil mapping products (29).

At the same time, machine learning techniques have seen a significant rise in remote sensing and geospatial data development (22). For instance, Homer et al., (30) used regression trees to create a categorical land cover map for the United States, while Coulston et al. (31) employed random forests to produce a continuous map of percent tree canopy cover. Additionally, various algorithms such as linear models, generalized additive models, artificial neural networks, support vector machines, normal Bayes, and k-nearest neighbor have been explored and tested (32, 33).

Although several studies have combined machine-learning models with model-agnostic explainability methods to explore links between urban environments and public sentiment (34, 35), the explicit quantification and spatial evaluation of predictive uncertainty in these areas remain largely underexplored. Existing research includes using random forests with Permutation Feature Importance (PFI) to identify park features associated with positive emotions (36) and applying LightGBM with SHapley Additive exPlanations (SHAP) to analyze interactions between geospatial features and sentiments (37). Other approaches involve multilevel regression, gradient boosting decision trees (GBDT) (8), and support vector regression (38) to examine how various urban factors influence sentiment. Despite these efforts, uncertainty is seldom visualized alongside model outputs and explanations, restricting clarity regarding the reliability of spatial predictions and their interpretations.

This study bridges the gap by combining predictive uncertainty with explainable modeling within a spatial sentiment framework. We train multiple machine-learning models to forecast sentiment based on urban environmental factors and assess location-specific uncertainty using Shannon entropy from predicted class probabilities. Using different classifiers together has been shown to be effective, uncovering spatial uncertainty among media sources through an entropy analysis. From a Digital Humanities perspective, a lack of transparency about algorithms’ outputs can raise questions regarding the method’s validity, affecting the analysis (39).

To understand the algorithms in this work, we utilize explainable AI (xAI), also called agnostic models. We employ SHapley Additive exPlanations (SHAP) to elucidate positive sentiment predictions, pinpointing the urban features that most influence the model’s outputs. Additionally, uncertainty maps can be integrated with SHAP explanations to identify areas where spatial predictions and interpretations are more dependable (lower uncertainty) and where caution is needed (higher uncertainty).

The methods described in this paper do not explain how the sentiment classifiers operate or their behavior. Instead, they suggest what features the underlying models might be considering or utilizing for their predictions. This approach enhances trust in the results from a Digital Humanities perspective, since the data is used to draw conclusions about the phenomenon represented in the case study. By linking uncertainty with interpretability, this method enhances the reliability of spatial inferences regarding sentiment and environmental interactions in Lisbon.

## 2 Materials and methods

**Figure 1:**
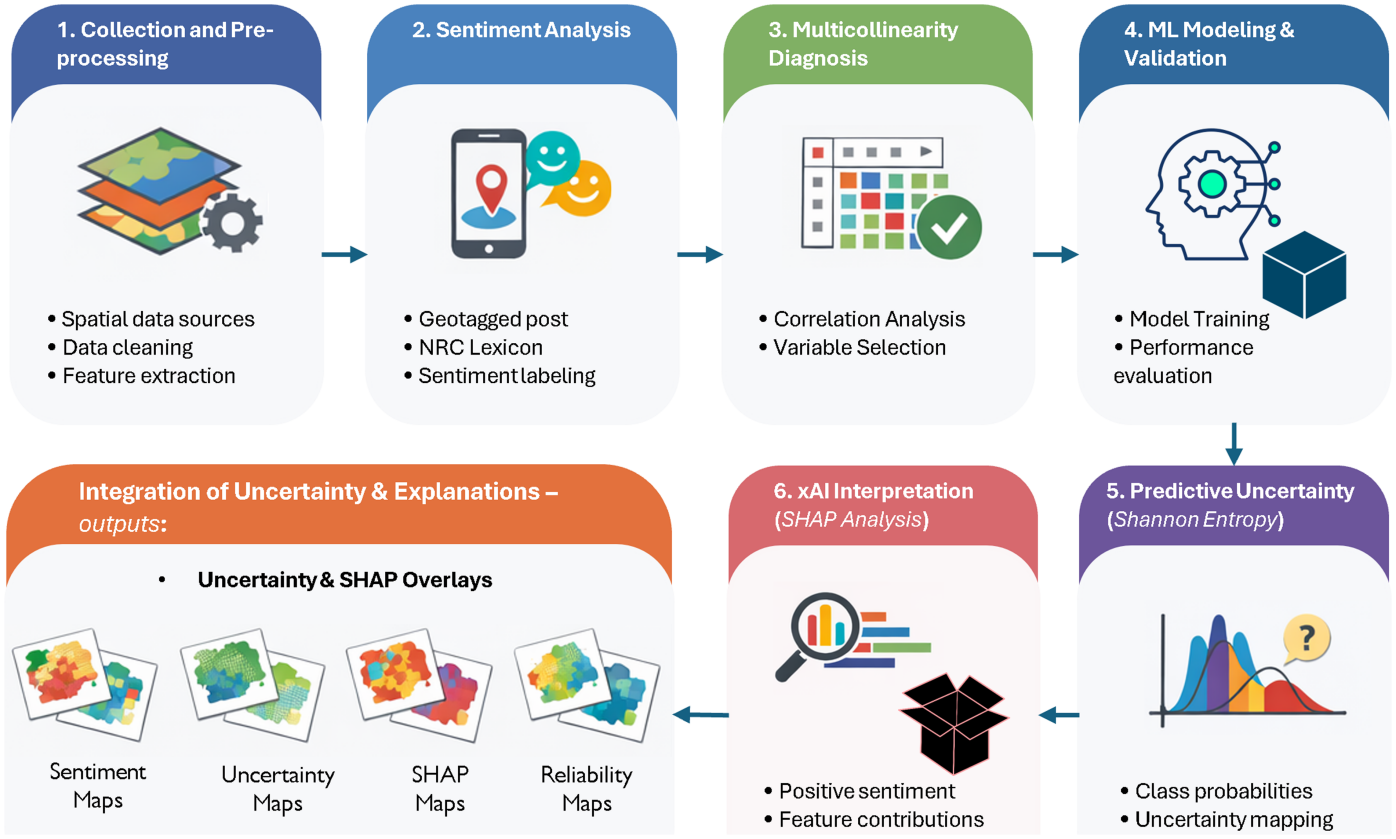
Methodology of the modelling process.

The methodological framework was developed to identify the urban environmental factors associated with sentiment in the city of Lisbon. It comprises six main phases: (1) collection and pre-processing of spatial data, (2) sentiment analysis, (3) diagnosis of multicollinearity among predictors, (4) development and validation of machine-learning models, (5) application of Shannon entropy to quantify prediction uncertainty, and (6) interpretation of model outputs using SHAP. The workflow is illustrated in Figure 1.

### 2.1 Sentiment analysis

Geographic and text-based analyses of social media data allow for the extraction of quantitative sentiment measures that are reliable and highly precise in space and time. Consequently, using social media to measure sentiment is now broadly acknowledged and accepted in academic circles (8). Among available platforms, Twitter (now X) is commonly selected due to its mixed-information content (42) and the relative ease of accessing and collecting data (43). X offers a large volume of near-real-time user-generated content, making it perfect for tracking public opinion across different topics (44–46). The choice of X instead of other platforms is also influenced by its wide public access to content and its easy-to-use interface, which supports the creation of data-extraction methods (47).

In the past, there were some concerns regarding the use of social media content in this context. These are mainly related to data quality, the post’s potential location inaccuracy, the representativeness of the population using social media, and possible biased behavior on social media (48).

Using social media data (49) demonstrated that there is a significant positive correlation between the seasonal patterns of visitors and hotel occupancy rates. Also, a study from (50) revealed a strong correlation between the number of Twitter users in each state and the 2010 US Census state populations.

Research utilizing social media has proven valuable for gaining deeper insights into city life, which is difficult to attain through other methods (51, 52). This approach offers advantages over traditional data sources, providing new opportunities beyond the limitations of costly, invasive cross-sectional surveys (53).

Sentiment Analysis seeks to automatically identify emotions and polarity in a text to classify its sentiment (54, 55). In this study, weuse a lexicon-based method with the NRC Emotion Lexicon (EmoLex), which links words and phrases to eight fundamental emotions (anger, fear, anticipation, trust, surprise, sadness, joy, and disgust), and two sentiment polarities (negative and positive). These associations are created through manual annotations gathered via crowdsourcing (56).

From the tweets published in Lisbon, only the georeferenced and public ones where users could provide exact GPS coordinates were selected. Initially, there were 16,791 georeferenced points from Twitter (now X) comments in Lisbon from 2019. Of these, only the comments made during daytime hours, between 9 a.m. and 7 p.m., summing 9,446 tweets, were included, as these hours represent peak urban space usage (57).

The approach to distinguish visitor tweets from local ones was based on the research conducted by (58). The authors measured the number of days between each user’s first and last uploaded comments. If this difference exceeded the average visit duration in Lisbon (2.1 nights), the tweets were classified as local. If it were shorter, the tweets would be categorized as belonging to visitors. For this analysis, we used the comments posted by local users in Portuguese.

Using the Portuguese version of EmoLex, each tweet was categorized according to specific emotions and sentiment polarities, creating a results table that was imported into a GIS system for visualization and spatial analysis of emotions and sentiments across the city. Out of these tweets, 1,919 matched the lexicon and were assigned a sentiment polarity label.

Lexicon-based approaches can offer distinct advantages over machine learning (ML) methods in sentiment analysis, particularly in specific contexts or languages like Portuguese from Portugal. Lexicon-based methods do not require extensive labeled training data, which is often scarce for less-resourced non-English languages. This makes them faster and more practical in such contexts (59–61).

The complexity of Portuguese creates challenges for sentiment analysis, necessitating specialized models for precise interpretation. This emphasizes a linguistic uniqueness that might be less evident in languages such as English (62, 63). In languages with limited NLP tools, such as Portuguese, lexicon-based methods can achieve competitive results. For instance, a Portuguese-specific lexicon demonstrated strong performance in sentiment analysis tasks (64).

For predictive modeling, the response variable (sentiment) was adjusted to fit a supervised machine-learning classification framework. Specifically, sentiment polarity was converted into a binary variable, with 0 indicating negative sentiment and 1 indicating positive sentiment.

### 2.2 Study area

**Figure 2:**
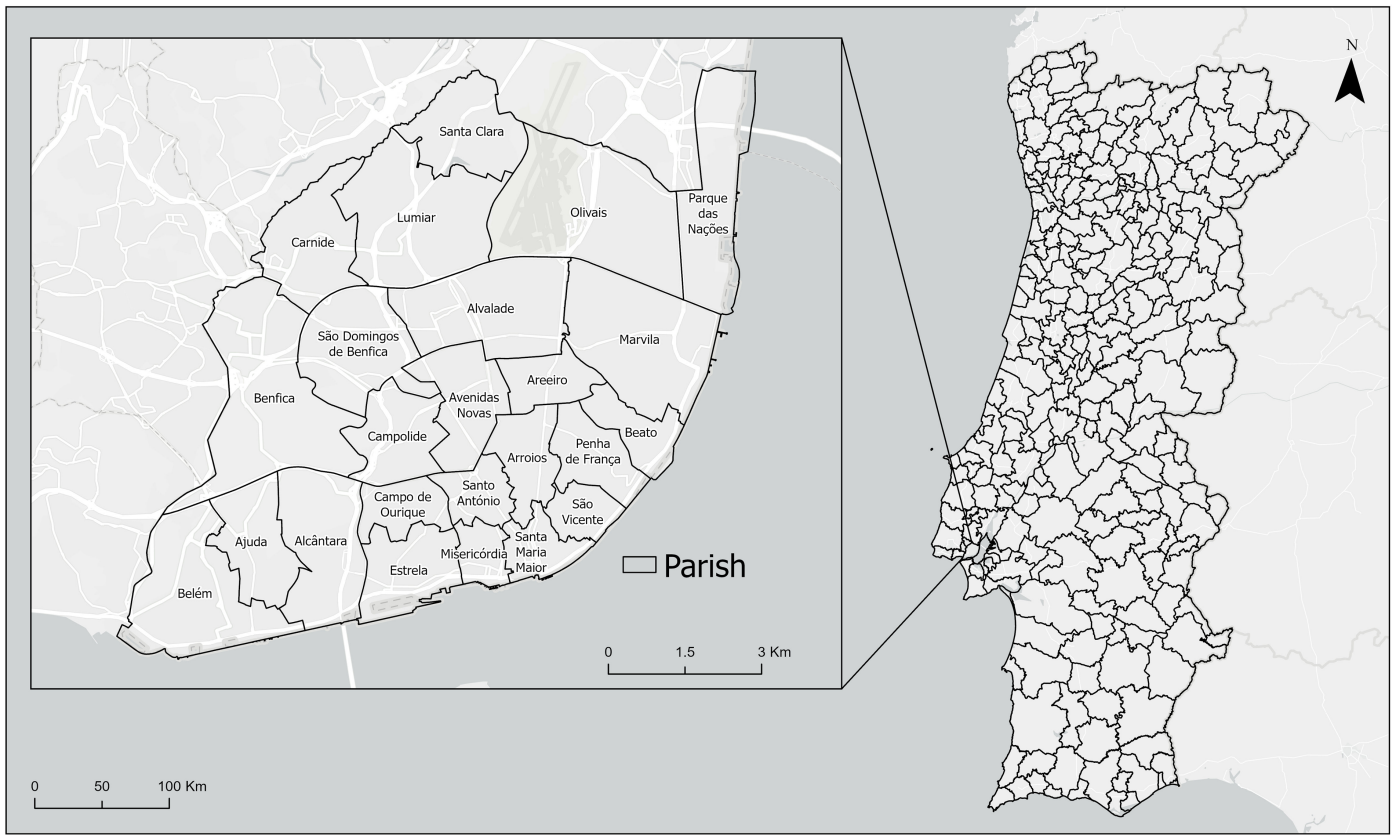
Study area: municipality of Lisbon (Portugal).

Lisbon, the capital of Portugal, is located on the northern bank of the Tagus River estuary. The municipality covers 86.83 km^2^, of which 70.24 km^2^ (≈81%) is classified as urbanized territory (40). Lisbon has 545,796 inhabitants (41), with a population density of 6,286 inhabitants per km^2^. Administratively, the municipality is subdivided into 24 parishes (Figure 2). Lisbon provides a relevant case study due to its diverse urban structure, combining historic areas, residential zones, and large green spaces such as Monsanto Forest Park. The city also presents spatial variability in environmental conditions, including differences in vegetation cover, accessibility to urban infrastructure, and exposure to air pollution and noise.

### 2.3 Selection of explanatory variables

The selection of the 30 independent variables was based on the conceptual framework of the relationship between urban form and mental well-being (65) and on the conceptual model of urban health-related well-being (UrbWellth) (66). Of all the variables, only those derived from the WorldView-2 satellite were originally found in raster format (2 m spatial resolution in multispectral bands). All other variables were subsequently converted to raster using these as references via distance, interpolation, and density operations (67).

The next stage involved a preliminary statistical analysis in which multicollinearity was assessed by calculating the variance inflation factor (VIF) (68, 69). VIF was computed in R software using a linear regression procedure to diagnose collinearity among the explanatory variables (70). Variables with a VIF greater than 5 were removed to reduce redundancy among predictors (71, 72). After calculating VIF and removing highly correlated variables, the model retained 10 explanatory predictors.

### 2.4 Machine learning models

The ML models were created using algorithms such as Random Forest (RF), Neural Networks (NNET), k-Nearest Neighbors (KNN), and XGBoost (XGB). The dataset was split into training (90%) and testing (10%) subsets. The training data was used to fit the models, while the test data were used to evaluate performance. Evaluation involved analyzing the confusion matrix to determine accuracy, sensitivity, specificity, positive predictive value, and negative predictive value.

The performance of four machine-learning models in sentiment prediction was assessed. Random Forest (RF) achieved the highest accuracy (0.82), while XGBoost (XGB) showed the lowest (0.71). Despite its comparatively lower predictive accuracy, XGB was still used in the follow-up analysis due to its compatibility with SHAP-based local explanations and its ability to support the visualization of aggregated Shapley values.

### 2.5 Shannon entropy

Shannon entropy was introduced in information theory to quantify uncertainty in a probability distribution (73). Higher entropy values signify increased uncertainty in the information. In our sentiment modeling framework, the RF and XGB classifiers generate predicted class probabilities for each pixel or location, ranging from 0 to 1. These probabilities can be employed to calculate Shannon entropy, providing a measure of predictive uncertainty in the generated sentiment maps (Equation 1):


$$H(X)=-\sum_{i=1}^{n}p(x_{i})*\log_{2}p(x_{i})$$ (1)

where H(X) denotes the entropy of the random variable X , n is the number of possible outcomes, and $p(x_{i})$ is the probability of outcome $x_{i}$ (73). The logarithm is base 2, so entropy is measured in bits (74).

Because sentiment is modeled as a binary outcome (positive vs. negative), with p=p(positive) and q=p(pegative)=1−p , entropy is given by (Equation 2):


$$H=-p\log_{2}(p)-q\;\log_{2}(q)$$ (2)

**Figure 3:**
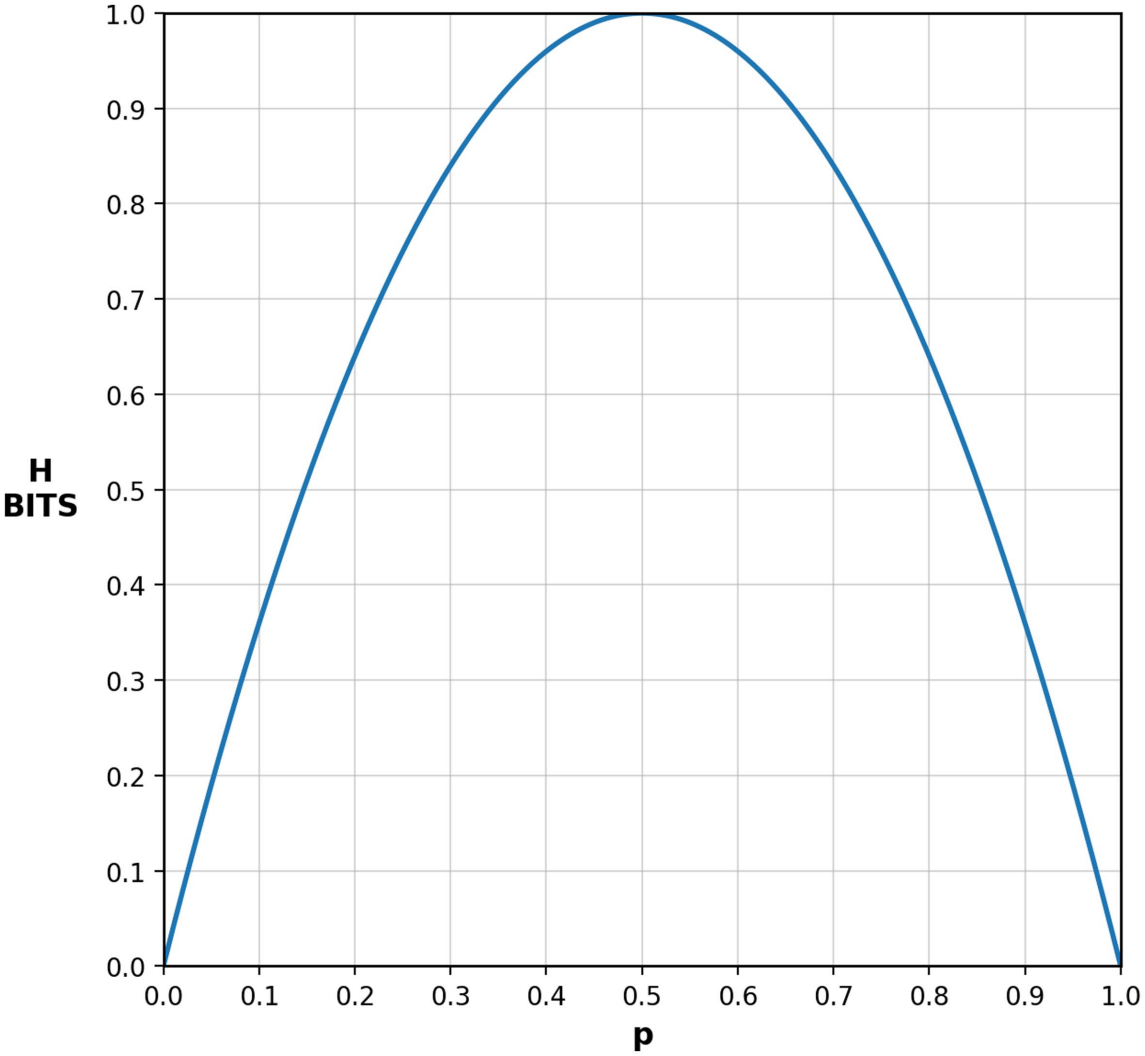
Shannon entropy for a binary outcome with probabilities *p* and 1 – 𝑝. Source: Shannon (73).

Entropy is minimal when p is close to 0 or 1 (high-confidence predictions) and maximal when p=0.5 , when both outcomes are equally likely and predictive uncertainty is greatest (Figure 3).

**Figure 4:**
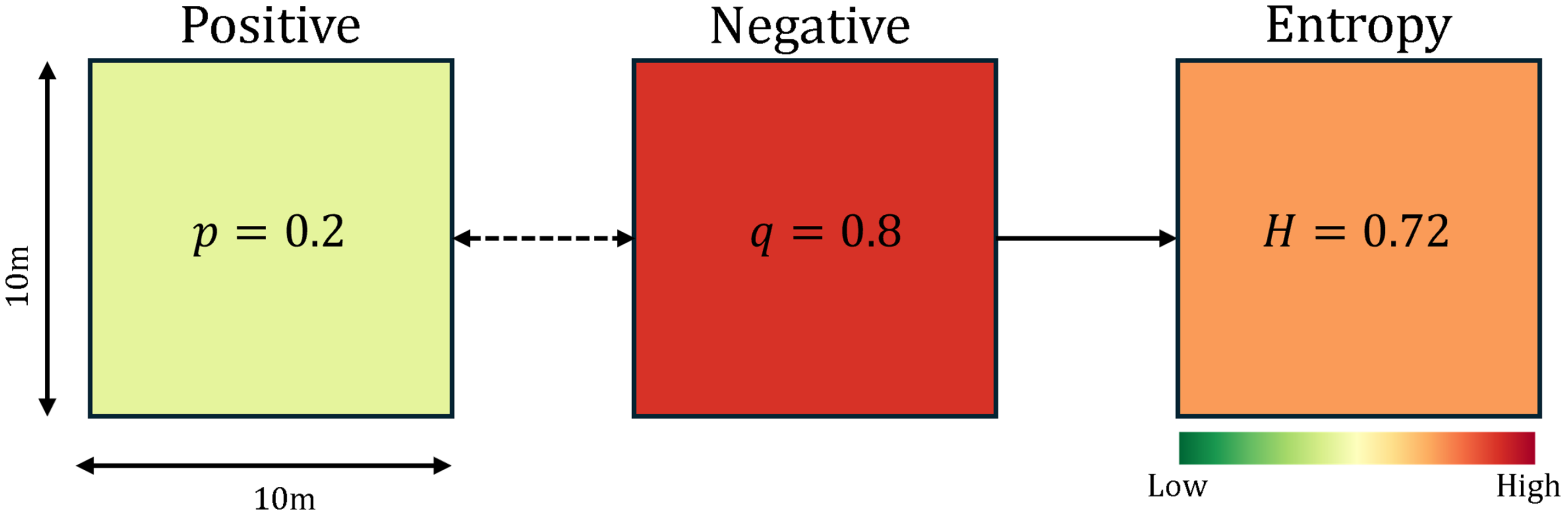
Pixel-level uncertainty assessment using predicted positive (*p*) and negative (𝑞 = 1 – *p*) sentiment probabilities and their corresponding Shannon entropy (𝐻).

We quantified uncertainty in the sentiment maps by using the binary Shannon entropy formula (Equation 2) on the predicted class probabilities at each pixel (see Figure 4). As previously mentioned, sentiment analysis is an NLP technique used to detect the emotional tone of a text and categorize it as positive or negative (75–77). In this context, each piece of text can be seen as a Bernoulli trial, where a random experiment produces just two possible outcomes, commonly called “success” and “failure,” such as positive or negative sentiment (78, 79).

In information theory, entropy indicates the uncertainty or unpredictability of a random variable. In Bernoulli trials, it reflects how hard it is to predict the outcome, making it a helpful measure of uncertainty in sentiment predictions (80). This leads to a specific form of entropy known as Binary entropy, which originates from information theory and quantifies the uncertainty or randomness in a binary variable. It is frequently used as a loss function in binary classification tasks like sentiment analysis. This function enhances the model’s predictions by penalizing mistakes and rewarding correct predictions, thus minimizing overall uncertainty (81, 82). The probabilistic perspective on binary cross-entropy helps estimate the chance of a specific sentiment, which is crucial for understanding the model’s variability.

Using binary entropy to evaluate sentiment prediction models involves measuring prediction uncertainty, optimizing with binary cross-entropy, and applying entropy-based techniques to enhance performance. This metric enables comparison among different sentiment analysis models, emphasizing which better handles uncertainty and produces more reliable predictions. Combining metrics like cross-entropy loss with accuracy, precision, recall, and F1-score provides a comprehensive view of the models’ predictive confidence and uncertainty (83).

This thorough assessment can uncover patterns of uncertainty unique to each model, assisting in choosing the most reliable sentiment analysis model. Moreover, it yields an entropy surface that highlights areas of higher predictive uncertainty, where sentiment classification should be interpreted with greater caution, and areas of lower uncertainty, where model outputs are more reliable (84, 85).

### 2.6 Explainable AI

Being able to accurately interpret a predictive model’s output is essential because it enhances user trust, offers insights for model improvement, and aids understanding of the modeled process. While simpler models like linear models are often chosen for their interpretability, the rise of big data has led to a preference for complex models, which increases the need to balance accuracy with interpretability. Since complex models are not easily understandable on their own, interpretation usually depends on an explanatory or simplified version of the original model (86).

Explainable AI (xAI), also known as model-agnostic modeling, is designed to interpret AI (and ML) decisions regardless of their complexity or internal structure, without requiring knowledge of how they function. Since an explainer should be capable of explaining any model, it must be model-agnostic (26). Model-agnostic methods can offer both overarching and specific explanations: overarching explanations outline what the model has learned from the input features, while specific explanations detail the reasoning behind individual predictions (12). Importantly, local fidelity does not necessarily mean global fidelity; variables that are important on a global scale might not be important locally, and the other way around (26).

In this study, we analyzed the XGBoost model by using SHapley Additive exPlanations (SHAP) with a model-agnostic Kernel SHAP method to evaluate how each explanatory variable influences the predicted outcome for individual observations (86). To support spatial analysis across Lisbon, SHAP values were visualized within a GIS platform and interpolated using Inverse Distance Weighting (IDW).

IDW is a well-known spatial interpolation technique, especially when the prediction depends on distance-based variables. It assumes that a known data point’s influence diminishes with distance, making it ideal for situations where proximity matters. The weights are inversely related to distance, so nearer points have greater influence on the results (87).

Furthermore, IDW can be improved by adding adaptive features, like distance-decay parameters that vary locally, which increase accuracy by considering spatial patterns and anisotropy in the data (88, 89). Using regression models or neural networks alongside, for example, improves predictions by combining distance-based weights with statistical trends, resulting in lower error rates than individual methods like Kriging (90).

Additionally, many predictor variables used in this study are distance-based, such as distance to green parks and sports facilities, and government official data, such as climate and pollution, are interpolated using IDW before being provided for public use.

The all process generated raster surfaces that highlight locations where certain factors have a greater impact on positive sentiment predictions. IDW proved its suitability for exploratory spatial analysis, while avoiding the imposition of statistical assumptions that may not be appropriate for SHAP-derived values. Such a workflow enables detailed, place-specific interpretation of the model outcomes and can guide targeted urban strategies.

#### 2.6.1 Shapley additive exPlanations

SHAP is a post-hoc explainability framework that assigns a model’s prediction to individual input features by leveraging Shapley values derived from cooperative game theory (19). More specifically, it applies the most commonly used solution concept for non-cooperative games (91), i.e., Nash equilibrium (NE) is a situation in a game where no player can improve their outcome by unilaterally changing their strategy, assuming all other players’ strategies remain the same. This concept can be applied to ensure that sentiment analysis models are stable and robust (92). By treating the interactions between various parts of the sentiment analysis system, such as different classifiers or feature extractors, as a game, it is possible to identify an equilibrium state where the system operates at its best without any component having a reason to change deviate.

In this context, SHAP helps distribute the total gains or costs among players accurately according to their contributions. Using Shapley values, it explains machine learning model predictions, enhancing transparency. SHAP is commonly employed to interpret model predictions by evaluating feature importance, which is vital for understanding and refining sentiment analysis models (93–95). In this case, for a given observation x , SHAP estimates how each feature contributes to the predicted outcome by distributing the “payout” (the model prediction) across the set of features in a theoretically grounded and additive manner (19).

Following the SHAP formulation, the explanation model is expressed as an additive feature-attribution model (Equation 3) (19):


$$\;g(z\prime )=\emptyset_{0}+\sum_{j=1}^{M}\emptyset_{j}z\prime_{j}$$ (3)

where g is the explanation model, $z^{\prime }\in {\left\{0,1\right\}}^{M}$ is a coalition vector indicating whether a feature is present, and $\emptyset_{j}$ , represents the Shapley value (feature attribution) for feature j . For the full coalition (all features present), the explanation simplifies to (Equation 4):


$$g(x\prime )=\emptyset_{0}+\sum_{j=1}^{M}\emptyset_{j}$$ (4)

In this study, SHAP values were estimated using a model-agnostic Kernel SHAP approach to explain the predicted probability of positive sentiment for each observation.

### 2.7 Software and libraries

R (version 4.2.3) and RStudio (2023.12.1) were used for sentiment analysis, machine-learning modeling, and explainability. Sentiment and emotion labels were derived using the syuzhet package, applying the NRC method based on the NRC Word–Emotion Association Lexicon.

Machine-learning models were developed using the caret package, which provides a unified workflow for training and evaluating predictive models (96). The XGBoost model was implemented using the xgboost package. Model interpretation was conducted using Kernel SHAP implemented with the shapr package. In addition, the pdp package was used to generate partial dependence plots for the Random Forest model.

## 3 Results

### 3.1 Sentiment prediction maps (RF vs. XGB)

**Figure 5:**
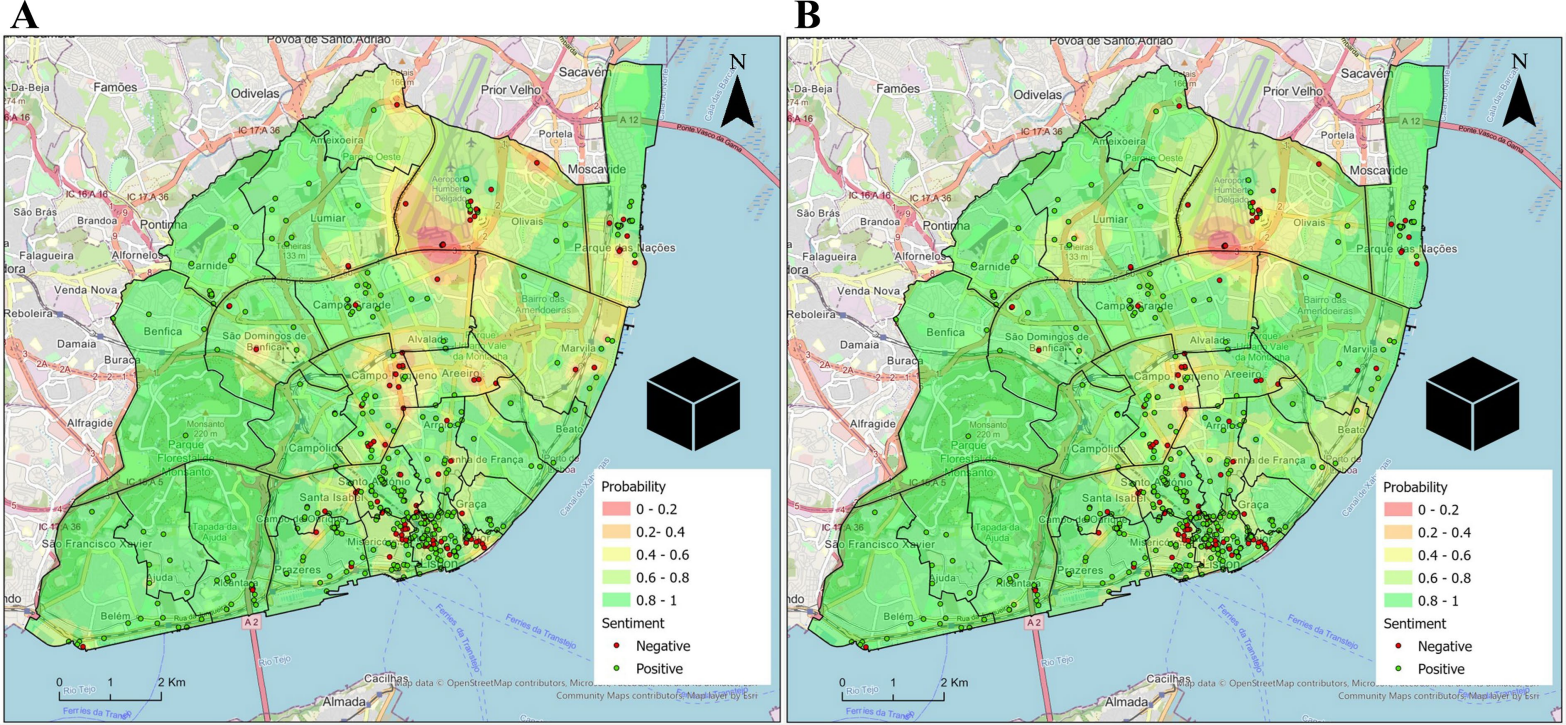
Probability of positive sentiment in RF model **(A)** and XGB model **(B)**.

Predicted positive sentiment probabilities from the Random Forest (RF) and XGBoost (XGB) models were visualized across Lisbon (Figures 5A,B). Higher values denote a greater likelihood of positive sentiment. In Figure 5A (RF), areas with high predicted probabilities generally align with locations labeled as positive by EmoLex (green points), indicating broad spatial consistency between the probability surface and lexicon-based labels. Lower probabilities appear around Lisbon Airport and residential zones. Figure 5B (XGB) shows a similar spatial pattern, with higher probabilities mainly in the southwest of the city. However, localized differences are evident, especially in Marvila and Parque das Nações, where the distribution of higher probabilities diverges from the RF surface.

### 3.2 Uncertainty analysis (entropy)

Using Shannon entropy on predicted class probabilities allows for quantifying predictive uncertainty and visualizing spatial areas of varying confidence. After applying Equation 2, the XGB model generally exhibited higher entropy than the RF model, aligning with its lower predictive performance shown in Table 1.

**Table 1:** Performance of ML algorithms in predicting sentiment.

Algorithm	Accuracy	Sensitivity	Specificity	Positive pred. value	Negative pred. value
RF	0.8229	0.7931	0.8358	0.6765	0.9032
NNET	0.7708	0.6552	0.8209	0.6129	0.8462
KNN	0.7865	0.7069	0.8209	0.6308	0.8661
XGB	0.7240	0.7183	0.7273	0.6071	0.8148

**Figure 6:**
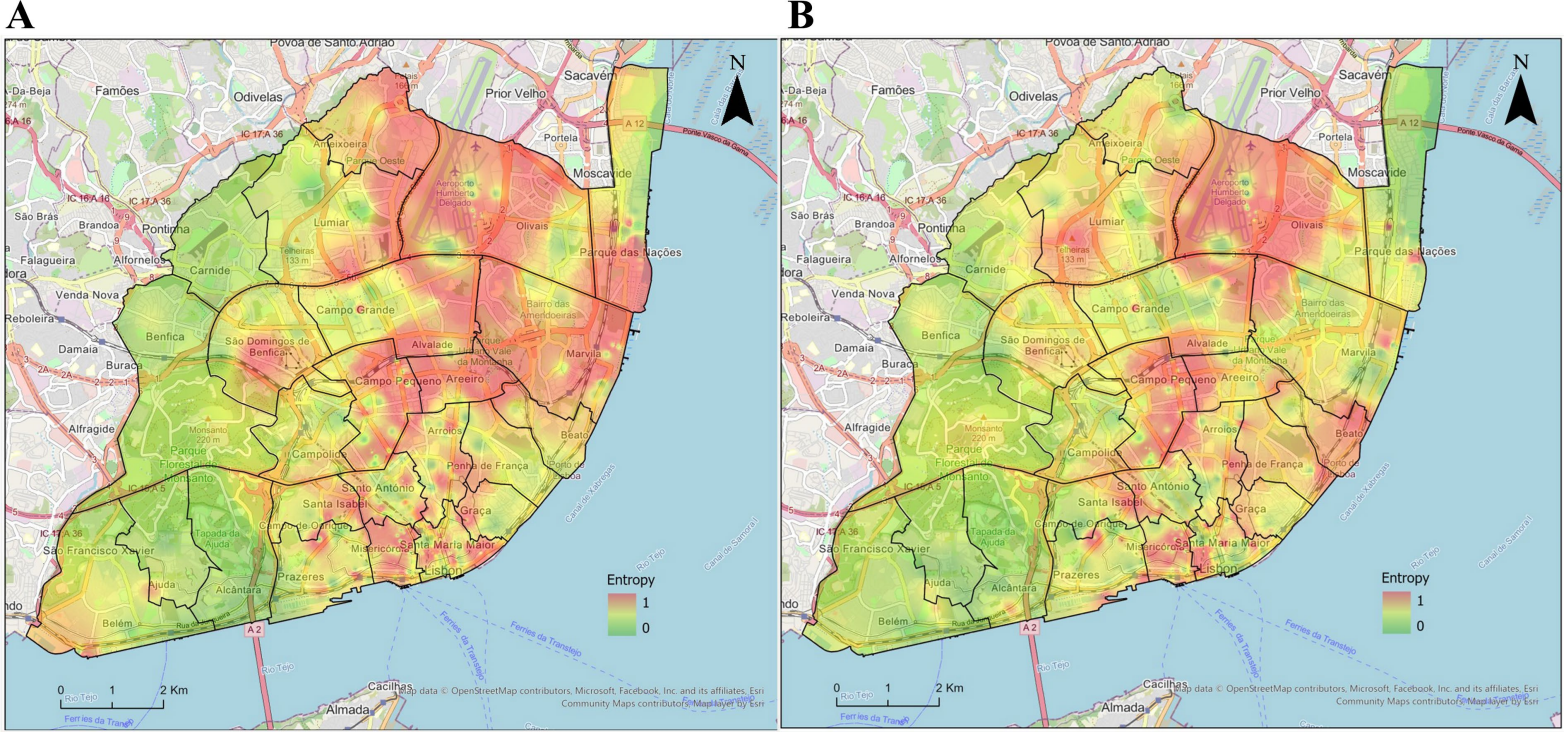
Entropy values for RF model **(A)** and XGB model **(B)**.

The entropy maps identify regions with higher uncertainty (greater entropy), where sentiment classification is less reliable. In the RF model (Figure 6A), the areas with the highest entropy are mainly in Misericórdia, Avenidas Novas, Areeiro, Marvila, Olivais, and Santa Clara. These zones should be approached with more caution when interpreting RF predictions. For the XGB model (Figure 6B), the highest-entropy zones are again in Misericórdia, Avenidas Novas, and Olivais, showing clusters where the model shows similar probabilities for both classes, indicating increased uncertainty. Conversely, in other parishes, the entropy levels are lower, pointing to more confident predictions with probabilities near 0 or 1.

### 3.3 Global explanations

**Figure 7:**
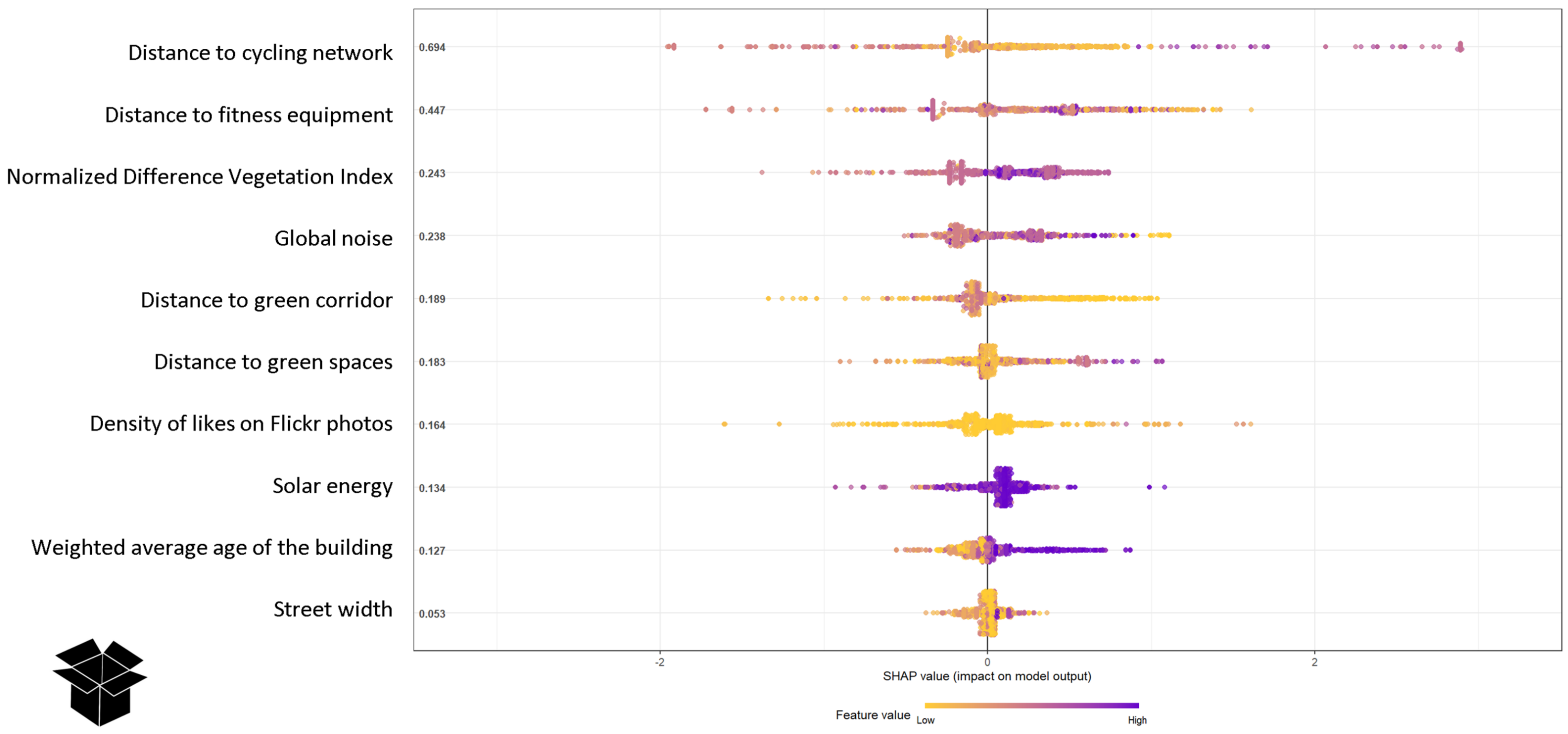
SHAP summary plot for the XGBoost model (XGBoost–SHAP).

Using the XGBoost model, we generated a SHAP summary plot for the sentiment dataset (Figure 7). The dependent variable is binary, derived from the EmoLex-based classification of posts, while the explanatory variables are continuous. Figure 7 can be interpreted as follows (97):

1. The *y*-axis lists the explanatory variables, ordered by importance (ranked by the mean absolute SHAP value);
2. The *x*-axis shows the SHAP value, i.e., the contribution of a feature to the predicted probability of the positive class;
3. The color gradient represents the original feature value (low to high). When high values (purple) cluster on the right, higher feature values tend to increase positive sentiment; when they cluster on the left, higher values tend to decrease it.

**Figure 8:**
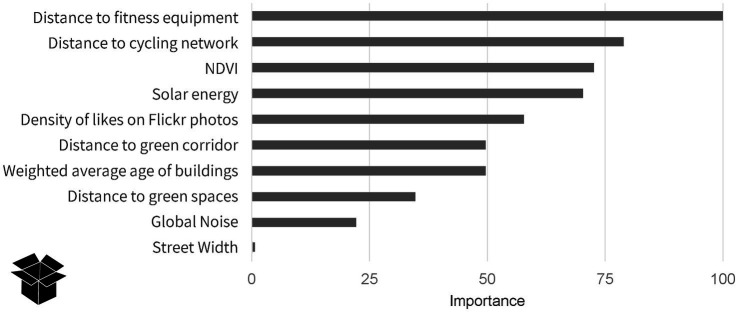
Most influential variables in explaining sentiment (PFI analysis) according to the RF model.

According to the XGBoost–SHAP framework (Figure 7), the most influential factors explaining positive sentiment in Lisbon in 2019 were the distance to cycling networks, the distance to fitness equipment, and the Normalized Difference Vegetation Index (NDVI). These results align with those from the Random Forest model using PFI (Figure 8). This consistency enhances confidence in the stability of the identified predictors, although the two interpretability methods offer different but complementary insights into how each variable influences model predictions.

### 3.4 Local explanations

**Figure 9:**
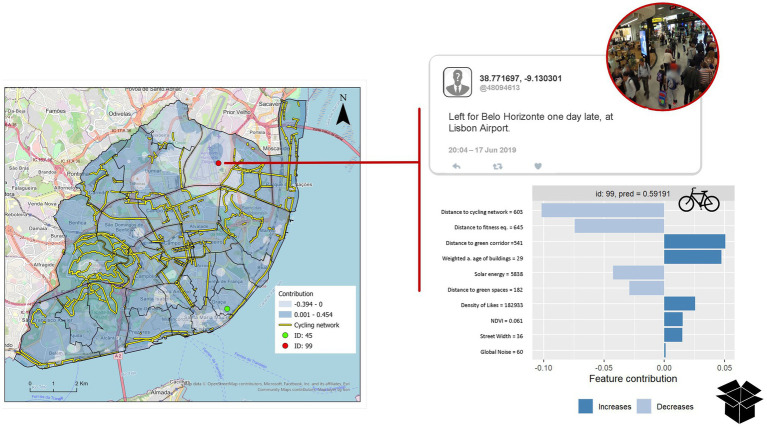
Contribution of distance to the cycling network to positive sentiment and Kernel SHAP explanation for observation 99 classified as positive sentiment.

Figure 9 shows the spatial mapping of Shapley-based explanations for Lisbon, emphasizing the variable distance to the cycling network. This map highlights regions where closer proximity to cycle lanes correlates with higher positive sentiment, while areas farther away tend to show negative contributions. Notably, negative contributions are observed around Humberto Delgado Airport and in Santa Maria Maior.

Figure 9 also illustrates the top ten most influential variables for observation 99, which shows a predicted probability of 0.59 for the positive class despite EmoLex labeling it as negative. In this case, the distances to cycling networks, fitness facilities, and green corridors have the largest absolute Shapley contributions. Notably, being 603 meters away from the cycling network lowers the likelihood of positive sentiment, while being 541 meters from green corridors increases it, as explained by the Kernel SHAP analysis. However, it is important to note that observation 99 is located in an area with high entropy, indicating higher uncertainty in the sentiment prediction. Therefore, caution is needed when interpreting the results for this location, as the model’s prediction is less reliable in regions with high uncertainty.

**Figure 10:**
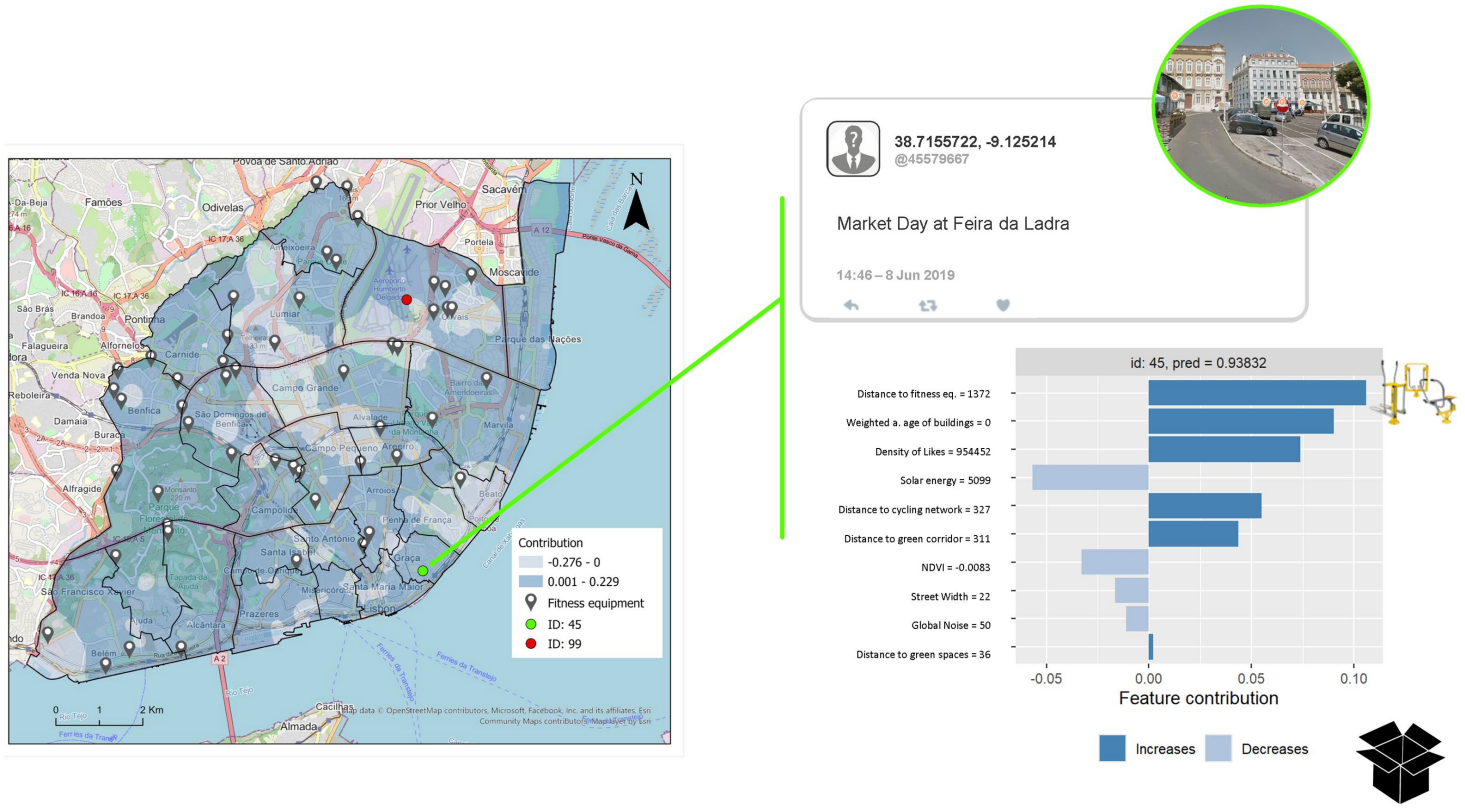
Contribution of distance to the fitness equipment to positive sentiment and Kernel SHAP explanation for observation 45 classified as positive sentiment.

Figure 10 illustrates the spatial distribution of Shapley-based explanations for the variable “distance to fitness equipment.” The findings reveal that this feature contributes both positively and negatively across different areas, without a clear overall pattern. Additionally, the figure presents the Kernel SHAP explanation for observation 45, which has a predicted probability of 0.94 for positive sentiment. In this case, the variables with the most significant Shapley contributions are distance to fitness equipment, weighted average of building age, and place popularity (density of likes on Flickr photos). Specifically, a distance of 1,372 meters to fitness facilities positively influences the likelihood of a positive prediction, while a solar energy value of 5,099 kW·m^−2^ has a negative impact. These SHAP values help clarify the model’s reasoning behind this prediction. Furthermore, observation 45 is located in an area with low entropy, which indicates higher certainty in the sentiment prediction. Therefore, the results for this observation can be considered more reliable.

**Figure 11:**
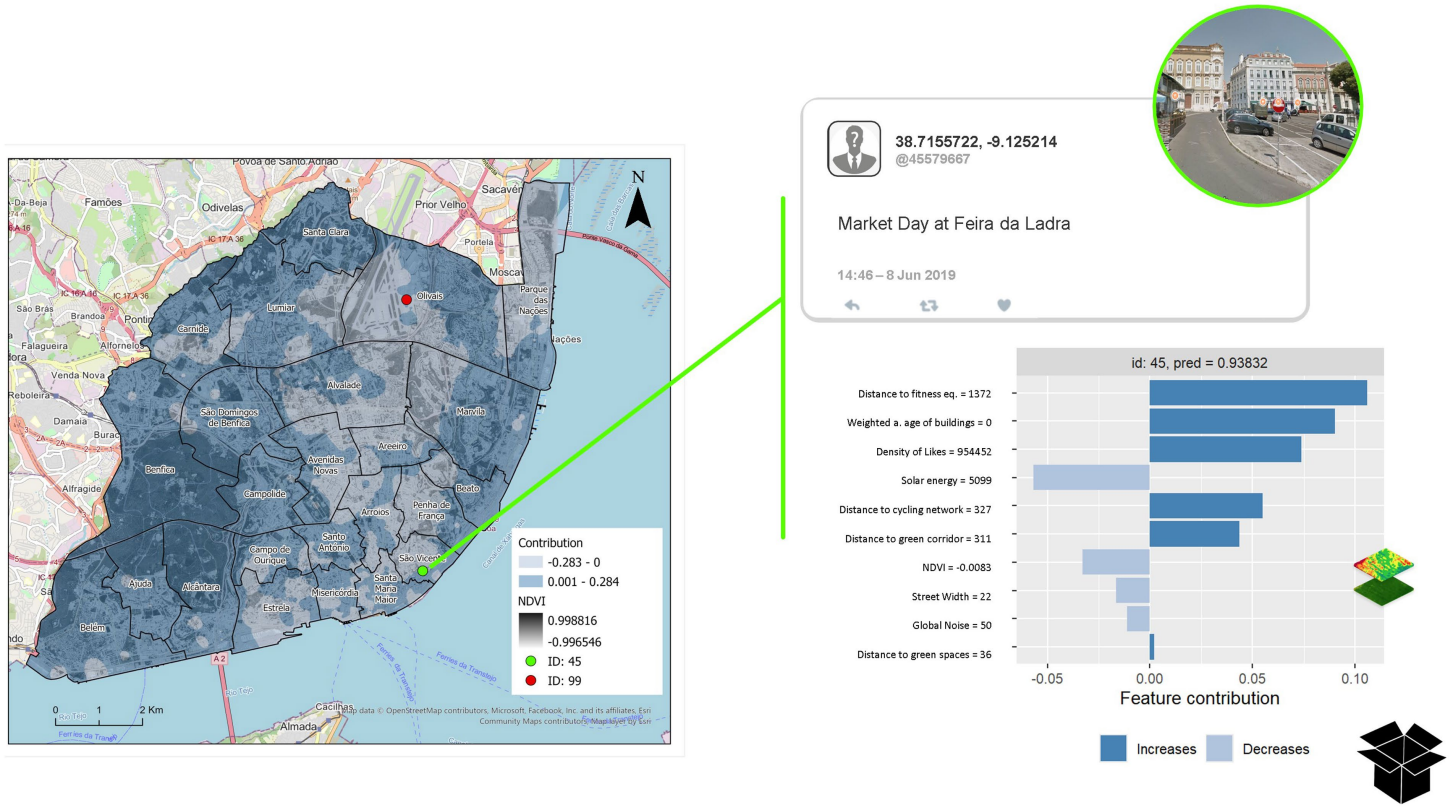
Contribution of NDVI to positive sentiment and Kernel SHAP explanation for observation 45 classified as positive sentiment.

As shown in Figure 11, analyzing NDVI-based Shapley explanations reveals the regions where vegetation significantly boosts the model’s predicted positive sentiment. Generally, higher NDVI values, which reflect denser vegetation such as in Monsanto Forest Park, positively influence sentiment. Conversely, areas with lower NDVI values, common in residential zones with minimal vegetation, tend to reduce the predicted positive sentiment. Figure 11 confirms this trend, indicating that an NDVI value of 0.0319 representing sparse or non-vegetated surfaces is linked to a decline in positive sentiment in the model’s output for this case.

## 4 Discussion

Generally positive sentiments prevail over negative ones. This comes in line to other works where sentiment analysis of online reviews in Portugal, such as on TripAdvisor and Twitter, indicated that most comments were positive, with prevalent emotions like joy and analytical responses. Negative feelings like anger or sadness were uncommon, possibly reflecting a more constructive or serious attitude toward online feedback (98).

The results indicate that proximity to cycling infrastructure and fitness facilities plays a significant role in shaping positive sentiments across Lisbon. These findings are consistent with previous studies highlighting associations between active mobility, physical activity and subjective well-being (99–101).

Cycling infrastructure is widely discussed as a lever for shifting urban travel toward active modes and reducing car dependence, particularly when implemented as connected, protected networks (102, 103). Evidence also suggests that protected cycling facilities are associated with lower injury risk compared with cycling in mixed traffic, supporting their relevance for perceived and objective safety (104). The SHAP results suggest that shorter distances to cycling infrastructure are generally linked to higher predicted positive sentiment, reinforcing the role of accessible active transport systems in enhancing urban well-being.

From an urban-planning perspective, incorporating sports and exercise infrastructure may support recreational activity, community engagement, and social interaction (105, 106). Access to fitness and sports facilities has been associated with higher levels of physical activity, supporting active lifestyles (107, 108). Regular exercise has also been associated with improved cardiovascular health, reduced risk of chronic diseases, and positive mental-health benefits, including reduced anxiety and improvements in mood and self-esteem (109). However, the spatial variability observed in SHAP contributions suggests that the relationship between fitness infrastructure and sentiment may be context-dependent, potentially reflecting differences in accessibility, quality, or surrounding urban conditions.

Vegetation, as captured by NDVI, emerges as a key factor influencing sentiment patterns. Higher NDVI values are associated with increased predicted positive sentiment, particularly in areas with dense vegetation such as Monsanto Forest Park. These findings are consistent with studies linking mental health and well-being with vegetation and positive sentiment (2, 110). One possible explanation is that NDVI may influence well-being through multiple pathways, including promoting physical activity and social interaction. However, it is important to note that NDVI captures overall vegetation coverage rather than accessibility or quality, and therefore may not fully reflect how green spaces are experienced or used by residents (110). Portuguese demonstrate unique emotional patterns in their comments. They tend to express positive feelings toward natural and ecological environments but are less detailed in their preferences than other linguistic groups (111). Furthermore, Portuguese social media users exhibit emotional intensity in their reactions to events, as in other cultures, although gender differences in emotional expression are less evident than in English-speaking contexts (112).

According to Rodrigues et al., in Lisbon, the distribution of environmental resources associated with well-being appears to be spatially uneven, while exposure to environmental risks is often concentrated in specific urban areas. Vegetation is often concentrated in large parks located outside the most central and densely built areas, while central districts tend to combine high accessibility with greater exposure to environmental stressors such as air pollution and noise, particularly in areas surrounding the airport (113).

More broadly, the findings of this study highlight the importance of integrating active mobility infrastructure and green environments in urban well-being. By combining machine learning predictions with uncertainty quantification and explainability techniques, this work provides a more transparent and spatially explicit framework for understanding sentiment–environment relationships. Such an approach can support more informed and evidence-based urban planning decisions, particularly in identifying areas where interventions may have the greatest impact.

## 5 Conclusion

This study demonstrates the potential of combining sentiment analysis, machine learning, uncertainty quantification, and explainable AI to better understand the relationship between urban environments and well-being. By integrating spatial prediction with entropy-based uncertainty measures and SHAP explanations, the proposed framework provides a more transparent and interpretable approach to analyzing sentiment patterns in urban contexts.

Across Lisbon, the results consistently highlighted proximity to cycling infrastructure and higher vegetation cover (NDVI) as prominent predictors associated with positive sentiment. These findings reinforce the relevance of active mobility and green environments in shaping urban sentiment, which is closely linked to subjective well-being. By promoting active lifestyles and enhancing access to nature through urban infrastructure, cities can create conditions that support a more favorable emotional environment for residents.

Moreover, the choice of sentiment-analysis approach is critical, because the labels assigned to posts directly shape the subsequent machine-learning pipeline. In this study, several locations were associated with elevated levels of negative sentiment in the mapped outputs (Figures 3–4). This pattern is plausibly influenced by limitations of lexicon-based labeling, which does not account for contextual meaning. Such mislabeling can propagate into model training and bias spatial interpretations. Future work should therefore explore alternative sentiment-classification approaches, including supervised and contextual language models, and compare how sensitive downstream spatial patterns are to the labeling method.

Finally, machine-learning models can support urban planners, policymakers, and public-health professionals by identifying patterns in large and heterogeneous urban datasets and translating them into actionable, spatially explicit insights. However, when model outputs are used to inform interventions, it is essential to report not only point predictions but also the uncertainty associated with them. Prediction uncertainty provides an explicit measure of confidence in each estimate, helping distinguish areas where results are comparatively robust from areas where additional data, validation, or cautious interpretation are needed. Incorporating uncertainty analysis into the workflow strengthens evidence-informed decision-making by clarifying the reliability of model-based recommendations in complex and dynamic urban settings. The use of spatial prediction, uncertainty mapping, and model-agnostic explanations increases the transparency of sentiment analysis, enabling more confident identification of areas where sentiment patterns and their drivers can be inferred.

## Data Availability

The raw data supporting the conclusions of this article will be made available by the authors, without undue reservation.
